# Supplementary figures and images for: Dual proteomics of Drosophila melanogaster hemolymph infected with the heritable endosymbiont Spiroplasma poulsonii
Source: PLoS One. 2021 Apr 29;16(4):e0250524. doi: 10.1371/journal.pone.0250524 (PMC8084229; doi:10.1371/journal.pone.0250524)

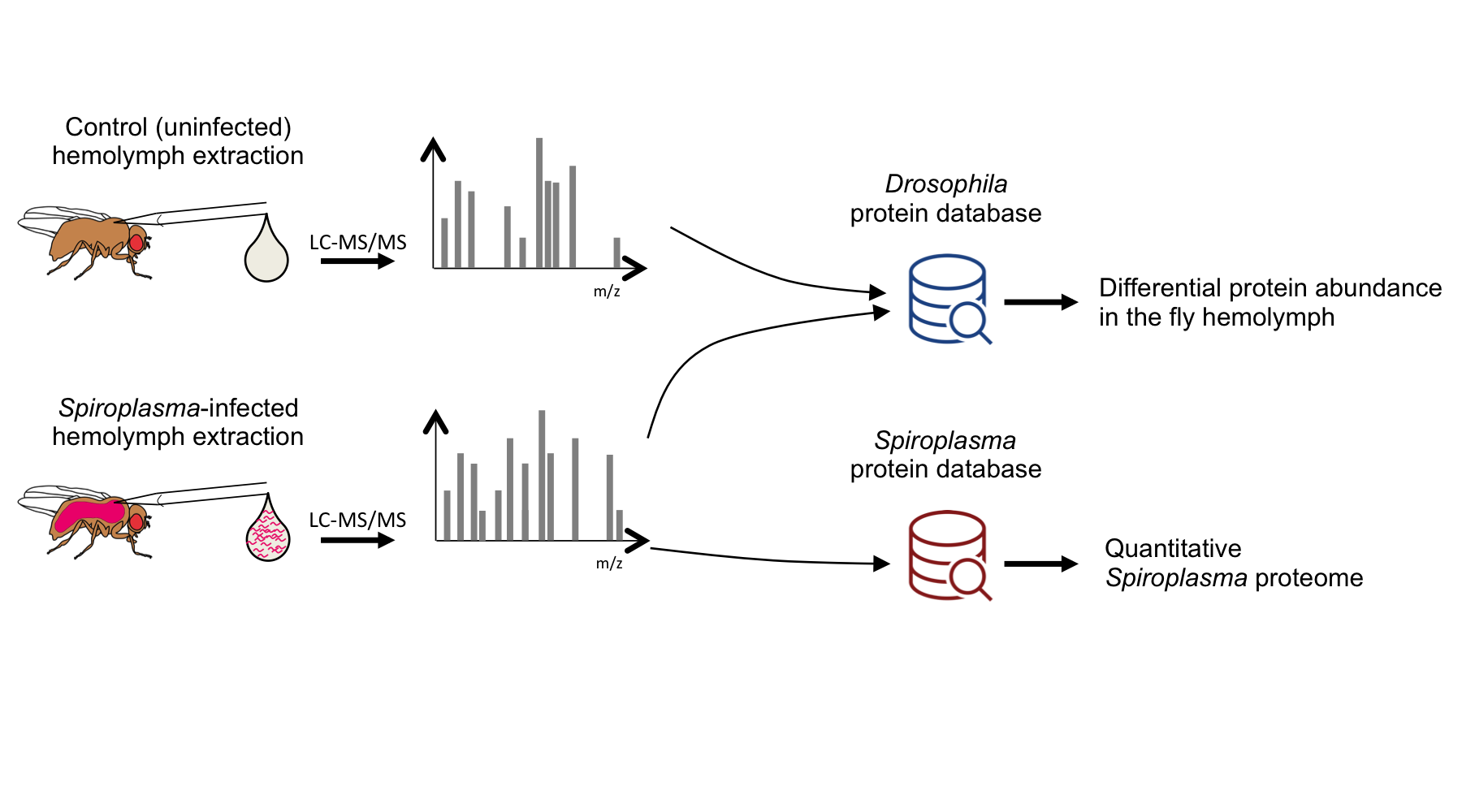

Supplement: S1 Fig — (TIF) [file pone.0250524.s001.tif]

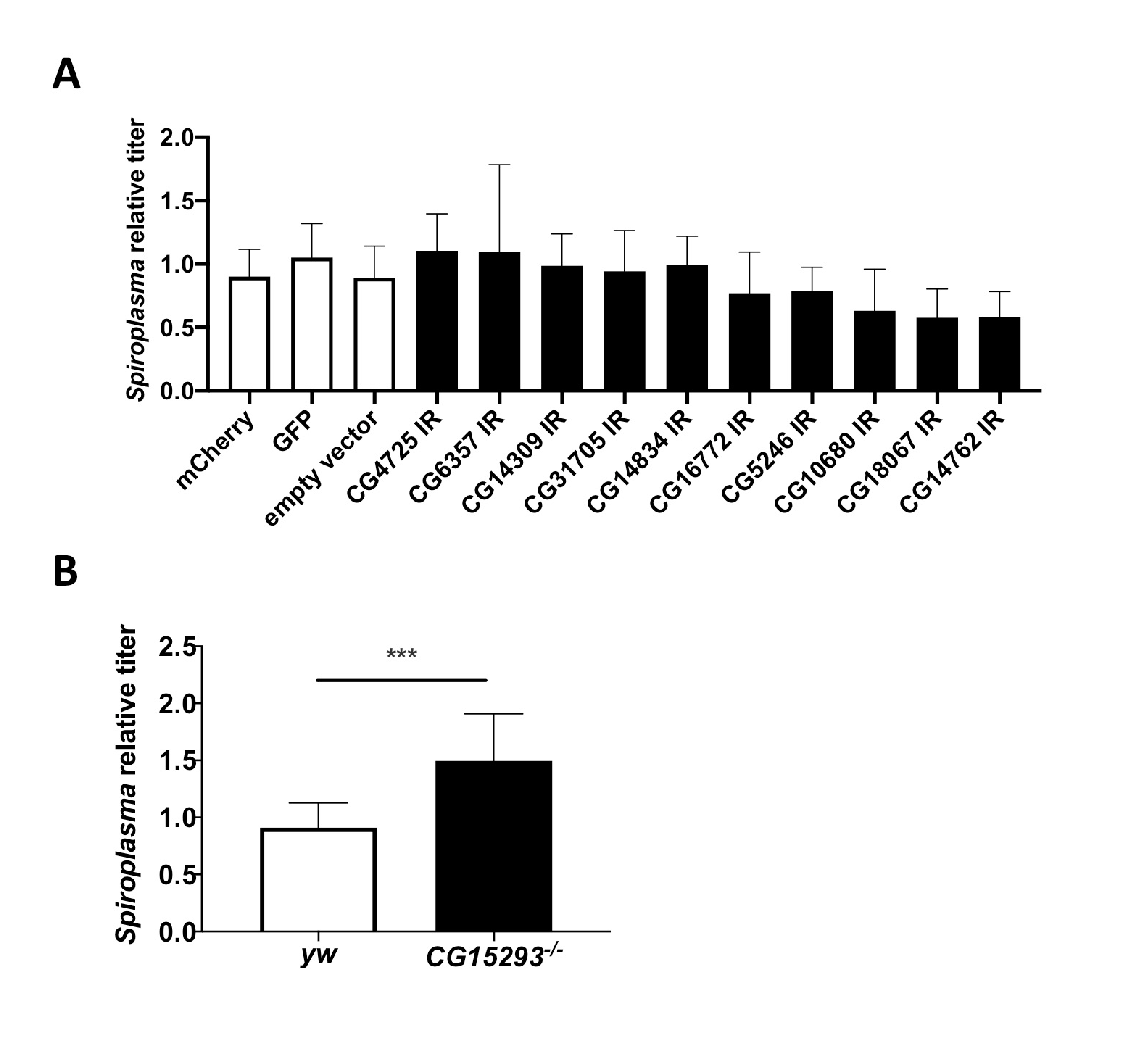

Supplement: S2 Fig — (A) Quantification of Spiroplasma titer in two weeks old flies. Titer is expressed as the fold-change of Act5C-GAL4>UAS-RNAi over Act5C-GAL4> w1118. Bars represent the mean +/- standard deviation of a pool of at least 2 independent experiments. (B) Quantification of Spiroplasma titer in CG15293 mutant flies compared to control yw flies. Bars represent the mean +/- standard deviation of a pool of at least 3 independent experiments. ***; p<0.0005 upon Mann-Whitney test on ΔΔCt values. (TIF) [file pone.0250524.s002.tif]
